# Supplementary material for: Combination of Optical Biopsy with Patient Data for Improvement of Skin Tumor Identification
Source: Diagnostics (Basel). 2022 Oct 15;12(10):2503. doi: 10.3390/diagnostics12102503 (PMC9600416; doi:10.3390/diagnostics12102503)
Supplement: Supplementary file 1 [file diagnostics-12-02503-s001.zip › diagnostics-1931276-supplementary.pdf]

Supplementary Material. PATIENT DEMOGRAPHICS

Figure S1 shows patient demographics of the experimental dataset.

617 spectral measurements of tumors were carried out for 615 patients of different ages including 178 men and 437 women who consulted specialized oncologists in Samara Regional Clinical Oncology Dispensary from May 2017 to December 2019.

The total spectral cohort includes 204 malignant tumors (70 malignant melanomas (MM), 122 basal cell carcinomas (BCC) and 12 squamous cell carcinomas (SCC)). As for gender distribution, 23 males and 47 females with MM, 50 males and 72 females with BCC, 5 males and 77 females with SCC are involved in study.

70 MM are distributed by location as follows: 5 MM in head and neck (h & n), 36 MM in trunk, 13 MM in upper limb, 13 MM in lower limb. 122 BCC are distributed by location as follows: 74 BCC in h & n, 34 BCC in trunk, 12 BCC in upper limb, 2 BCC in lower limb. 12 SCC are distributed by location as follows: 7 SCC in h & n, 4 SCC in trunk, 1 SCC in upper limb.

Mean ages of patients with melanoma, BCC, and SCC are 58 (25–84) years, 68 (26–87), 70 (58–84) correspondingly.

Among 413 benign tumors 26 dermatofibromas (DF), 62 papillomas (PP), 40 hemangiomas (HE), 113 seborrheic keratosis (SK), 170 nevi (NE) (all types), 1 cutaneous horn and 1 benign tumor of epidermal appendage are observed. 100 males and 313 females with benign tumors were involved in our study including 32 males and 138 females with pigmented NE, 32 males and 81 females with SK.

In total, 106 benign tumors are registered in h & n, 209 in trunk, 44 in upper limb, and 54 in lower limb. 170 pigmented NE are distributed by location as follows: 36 NE in h & n, 36 NE in trunk, 13 NE in upper limb, 13 NE in lower limb. 113 SK are distributed by location as follows: 40 SK in h & n, 53 SK in trunk, 9 SK in upper limb, 11 SK in lower limb.

Mean age of patients with benign tumors is 60 (20–86) while for NE cases mean patient age was 44 (18–86) and for SK – 62(21–84)

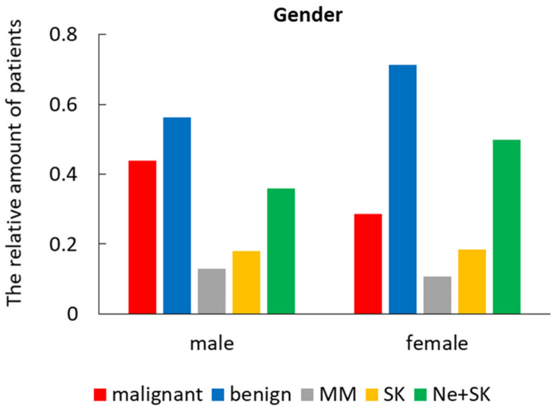

(a)

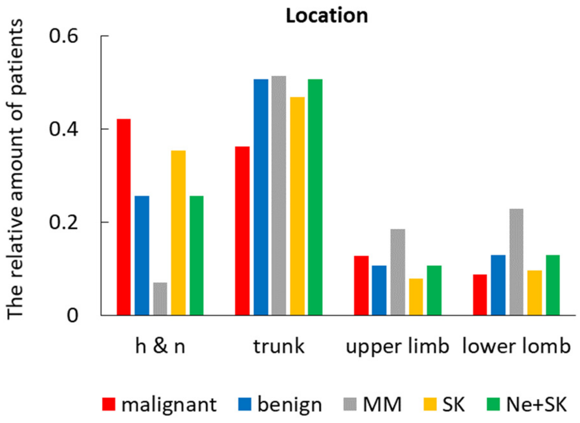

(b)

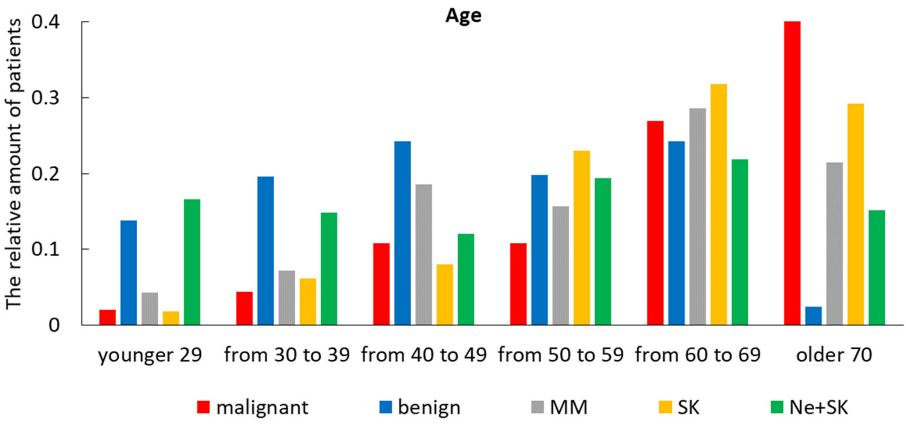

(c)

Figure S1 – Patients' statistics: (a) distribution by gender; (b) distribution by localization; (c) distribution by age groups
